# Population structure, genetic diversity and downy mildew resistance among *Ocimum* species germplasm

**DOI:** 10.1186/s12870-018-1284-7

**Published:** 2018-04-23

**Authors:** Robert M. Pyne, Josh A. Honig, Jennifer Vaiciunas, Christian A. Wyenandt, James E. Simon

**Affiliations:** Department of Plant Biology, Rutgers, the State University of New Jersey, Foran Hall, 59 Dudley Rd, New Brunswick, NJ 08901 USA

**Keywords:** *Ocimum* spp., Downy mildew resistance, EST-SSRs, Population structure, Genetic diversity, Polyploid

## Abstract

**Background:**

The basil (*Ocimum* spp.) genus maintains a rich diversity of phenotypes and aromatic volatiles through natural and artificial outcrossing. Characterization of population structure and genetic diversity among a representative sample of this genus is severely lacking. Absence of such information has slowed breeding efforts and the development of sweet basil (*Ocimum basilicum* L.) with resistance to the worldwide downy mildew epidemic, caused by the obligate oomycete *Peronospora belbahrii*. In an effort to improve classification of relationships 20 EST-SSR markers with species-level transferability were developed and used to resolve relationships among a diverse panel of 180 *Ocimum* spp. accessions with varying response to downy mildew.

**Results:**

Results obtained from nested Bayesian model-based clustering, analysis of molecular variance and unweighted pair group method using arithmetic average (UPGMA) analyses were synergized to provide an updated phylogeny of the *Ocimum* genus. Three (major) and seven (sub) population (cluster) models were identified and well-supported (*P* < 0.001) by PhiPT (Φ_PT_) values of 0.433 and 0.344, respectively. Allelic frequency among clusters supported previously developed hypotheses of allopolyploid genome structure. Evidence of cryptic population structure was demonstrated for the k1 *O. basilicum* cluster suggesting prevalence of gene flow. UPGMA analysis provided best resolution for the 36-accession, DM resistant k3 cluster with consistently strong bootstrap support. Although the k3 cluster is a rich source of DM resistance introgression of resistance into the commercially important k1 accessions is impeded by reproductive barriers as demonstrated by multiple sterile F1 hybrids. The k2 cluster located between k1 and k3, represents a source of transferrable tolerance evidenced by fertile backcross progeny. The 90-accession k1 cluster was largely susceptible to downy mildew with accession ‘MRI’ representing the only source of DM resistance.

**Conclusions:**

High levels of genetic diversity support the observed phenotypic diversity among *Ocimum* spp. accessions. EST-SSRs provided a robust evaluation of molecular diversity and can be used for additional studies to increase resolution of genetic relationships in the *Ocimum* genus. Elucidation of population structure and genetic relationships among *Ocimum* spp. germplasm provide the foundation for improved DM resistance breeding strategies and more rapid response to future disease outbreaks.

**Electronic supplementary material:**

The online version of this article (10.1186/s12870-018-1284-7) contains supplementary material, which is available to authorized users.

## Background

The genus *Ocimum* is estimated to contain approximately 64 herbaceous annual and perennial plant species [1]. The primary center of diversity is described as being in Tropical Africa [2], while a secondary center exists in Tropical Asia and a tertiary center exists in the New World tropics [2, 3]. A number of species are cultivated and processed throughout the world for application in medicinal, dietary supplement, essential oil, food flavoring and culinary industries [4]. *Ocimum basilicum* L., sweet basil, is among the most economically important culinary herbs in the United States, Europe and Israel [5]. Plant breeding efforts have largely targeted sweet basil for improved disease resistance [6], chilling tolerance [7] and novel volatile profiles (chemotypes) [8].

*Ocimum* spp. demonstrate relatively high rates of outcrossing in the presence of pollinators [9], while readily self-pollinating in their absence. The flexibility of this reproductive system has been exploited by natural and artificial selection, likely serving as a catalyst for morphological and volatile diversification of *Ocimum* spp. [4]. In addition, cytological investigation suggests this genus has also undergone extensive genome augmentation resulting in ploidy variation. While the x = 12 basic chromosome number is considered stable in tetraploid (2n = 4× = 48) *O. basilicum*, different ploidies have been reported across other *Ocimum* species [10]. Dual basic chromosome numbers of 8 and 12 have been proposed for two major ‘Basilicum’ and ‘Sanctum’ groups, respectively [11]. More recently, studies involving nuclear DNA content support the hypothesis of x = 12 as the genus-wide basic chromosome number with some cases of aneuploidy [12, 13]. This explanation accounts for dysploidy, which has proven to be a persistent phenomenon in plant evolutionary biology [14]. The 386 Mbp *O. tenuiflorum (*syn. *O. sanctum*) genome was reported to be a diploid (2n = 2× = 16) supporting the existence of an x = 8 basic chromosome number and suggesting *O. tenuiflorum* is a diploid species [15]. Thus, competing theories of major genome structure remain prevalent in the *Ocimum* genus.

Examination of chromosome pairing behavior suggests allopolyploidy likely explains the polyploid genome structure in basil [10, 11]. For instance, karyological investigation of *O. basilicum* and *O. basilicum* x *O. americanum* F_1_ hybrids found no evidence of tetravalent and little trivalent formation (4-8% of pollen mother cells) demonstrating high levels of preferential pairing within sub-genomes [11]. Relatively high levels of inter-specific hybridization [9] suggest allopolyploid formation to be more likely than autopolyploidy [16]. This hypothesis is also supported by genotype data from simple sequence repeat (SSR) and single nucleotide polymorphism (SNP) markers in the *O. basilicum* MRI x SB22 F_2_ mapping family demonstrated that disomic segregation providing molecular evidence for an allotetraploid (AABB) *O. basilicum* [17].

In addition to variation in ploidy [10, 18], a variety of factors including unknown geographic origin and phenotypic diversity complicate classification of relationships among *O. basilicum* and the greater *Ocimum* genus. Taxonomical incongruencies are also widespread throughout the literature and among seed distributors. Furthermore, absence of standardized descriptors or voucher specimens for particular cultivars has been particularly problematic in classifying *O. basilicum* [1]. Species of economic salience such as *O. x citriodorum*, *O. americanum* and *O. basilicum* var*. citriodorum* are cited interchangeably throughout the literature making comparison across studies challenging [19]. Despite attempts to standardize nomenclature [19, 20] the species epithets continue to be used redundantly.

Morphological characters and volatile composition (chemotype) have been used to estimate genetic diversity in the *Ocimum* genus [21–24]. Classification based on phenotypic characters is problematic in that homologous similarities may be difficult or impossible to discriminate from traits resulting from convergent evolution [25]. Reliance upon analyses using these metrics may be particularly damaging in the context of plant breeding, which relies heavily upon genetic relationships among germplasm to make informed decisions with regard to selection and crossing decisions [26]. Nevertheless, the most comprehensive study of *Ocimum* to date remains that of Paton et al., which relied exclusively upon morphological characters [1]. Results of this study delineated three major divisions in the genus or subgenera corresponding into *Ocimum*, *Nautochilus* and *Gymnocium* [1]. Subgenera *Ocimum* is further divided into three sections including *Gratissima*, *Hiantia*, and *Ocimum*, where *O. basilicum* and *O. americanum* was placed [1].

Molecular phylogenetic approaches have employed RAPD and/or AFLP markers to determine relationships among nine [27], twelve [28], twenty-two [18], twenty-eight [12] and thirty seven [2] *Ocimum* spp. accessions. DNA markers from three plastid regions were used by Paton et al. to classify 12 *Ocimum* spp. accessions within the Lamiaceae family [3]. More recently, inter-simple sequence repeat (ISSR) markers were found to have higher polymorphism information content (PIC) and greater resolving power than chloroplast DNA (cpDNA) *psbA-trnH* markers following cluster analysis of 12 accessions representing 8 *Ocimum* spp. [29]. Despite being the preferred marker systems for phylogenetic analyses, neither SSR nor SNP markers have been used to classify relationships among *Ocimum* spp. accessions. SSRs are considered advantageous because they are multi-allelic, polymorphic, reproducible, ubiquitous throughout the genome and amenable to high-throughput genotyping with multiplexed PCR [30]. Expressed sequence tag (EST) derived SSRs (EST-SSRs) are particularly desirable for genetic diversity and mapping studies due to their location in coding regions and transferability across related germplasm [31, 32]. The National Center for Biotechnology Information (NCBI) *O. basilicum* EST library was recently used to develop polymorphic SSRs and genotype the MRI x SB22 mapping population [17].

The deficit of available sweet basil genetic and genomic resources relative to other cultivated plant species has been highlighted in recent years by a worldwide downy mildew (DM) epidemic caused by the oomycete pathogen *Peronospora belbahrii* [33, 34]. As demonstrated by extensive breeding across plant species affected by downy mildew, deployment of genetic resistance is an essential management strategy for effective disease control [35–37]. Recent reports of Mefenoxam resistant *P. belbahrii* isolates in Israel [38] and Italy [39, 40] exacerbate the need for resistant cultivars. However, development of resistant sweet basil varieties has been dramatically slowed by a severe lack of knowledge regarding genetic similarity between elite *O. basilicum* and exotic *Ocimum* spp. germplasm rich in sources of resistance [41–43]. This has rendered attempted introgression of resistant loci into sweet basil accessions subject to rudimentary trial and error by laborious cross-pollinations between elite and exotic breeding lines. Among 27 F_1_ hybrids evaluated by Ben-Naim et al., those considered ‘highly resistant’ were completely sterile [41].

Population structure is among the analyses most needed to better elucidate relationships among *Ocimum* spp. Model-based clustering is a powerful analysis method frequently used to infer association of individuals into distinct populations from multilocus data sets [44]. Association of germplasm into distinct populations provides essential a priori information needed to avoid issues of sterility while monitoring potential narrowing of the genetic pool among breeding populations. Furthermore, detection of cryptic population structure is an important prerequisite to association mapping (AM) of alleles with traits of interest (i.e. disease resistance) by avoiding false positive associations [44, 45]. Identification of population structure and estimation of genetic diversity among *Ocimum* spp. is needed to provide a more robust breeding response to future biotic or abiotic stresses.

In this study no designations of species/relationships are considered to provide an unbiased update of basil phylogenetics and first insights into population structure. We hypothesized that SSR markers developed from the available NCBI EST database could be employed to increase resolution of genetic relationships among a diverse panel of 180 *Ocimum* spp. accessions by population structure and genetic diversity analyses. Evaluation of these accessions for response to downy mildew provides insight into the distribution of downy mildew resistance within the *Ocimum* genus and has important implications for disease resistance breeding strategies.

## Results

### Response to *Peronospora belbahrii*

Significant differences (*P* < 0.001) were detected among 180 genotypes evaluated for response to DM (Additional file 1). Eighty-two accessions (46%) demonstrated a mean DS < 1.0 (considered resistant) of which 33 (18%) exhibited no sporulation (DS = 0). A large proportion (17%) of the accessions with mean DS < 1.0 were Rutgers breeding lines with MRI-conferred DM resistance (DM_MRI_). DM susceptibility was widespread, however, with 71 accessions (40%) having DS ≥ 3.0 (considered highly susceptible). The remaining 26 accessions (15%) were in an intermediate range of DS between 1 (10%) and 3 (50%) representing levels of DM tolerance. A single accession, PI 511865, segregated for response to DM indicating heterozygosity among loci controlling resistance in this accession.

### EST-SSR polymorphism

The 20 EST-SSR markers used in this study were pre-screened for unambiguous PCR amplification and demonstrated a low percentage of null genotypes. A list of DNA sequence and melting temperatures (T_m_) associated with EST-SSR markers can be found in Additional file 2. A minimum of 90% and an average of 96% of individuals were successfully genotyped across all EST-SSR markers. A total of 269 unique alleles were amplified across the 180 *Ocimum* spp. panel and 2 outgroup *Nepeta cateria* accessions (CN3 and CR9). The average PIC across 20 EST-SSR markers was 0.14, ranging from 0.01 to 0.48 and 6 to 24 alleles per marker (locus) (Table 1). The average number of alleles/locus across the full panel was 1.86 (Table 2).

**Table 1:** Description of 20 EST-SSR markers used to classify 180-accession panel of *Ocimum* spp.

MarkerID	Genbank/Contg ID	Repeat Motif	Alleles	PIC	Product Size	LG	Position (cM)
OBNJR2sg33	DY333933	(AC)16	19	0.01-0.45	271-299	14	82.7
OBNJR2cn29	Contig1138	(AC)16	11	0.02-0.45	246-448	13	54.6
OBNJR2sg04	DY343638	(GA)17	27	0.01-0.48	268-357	13	40.2
OBNJR2sg30	DY336727	(AG)22	20	0.01-0.42	241-279		
OBNJR3sg124	DY331703	(GCC)6	11	0.01-0.40	161-327		
OBNJR3sg19	DY343509	(TCA)6	6	0.01-0.46	195-432	6	18.9
OBNJR3cn298	Contig2510	(CTA)6	9	0.01-0.42	298-322		
OBNJR3cn359	Contig2911	(GGC)6	11	0.01-0.31	161-292		
OBNJR3cn362	Contig2969	(TGA)6	7	0.02-0.45	225-243	3	78.0
OBNJR3sg155	DY325572	(GTT)7	8	0.02-0.40	188-263		
OBNJR3sg168	DY323726	(GAA)7	11	0.01-0.44	297-390		
OBNJR3sg113	DY335879	(CCT)7	12	0.01-0.32	313-392		
OBNJR3cn56	Contig582	(AGG)7	10	0.01-0.45	169-240	3	84.7
OBNJR3cn74	Contig715	(CAG)7	12	0.01-0.43	211-244		
OBNJR3sg145	DY328393	(GCT)8	15	0.01-0.45	271-313		
OBNJR3cn03	Contig100	(CCA)10	15	0.01-0.44	198-262		
OBNJR3cn210	Contig1890	(AAG)11	14	0.01-0.43	266-302		
OBNJR3cn240	Contig2142	(ATA)16	24	0.01-0.37	254-370	19	88.7
OBNJR3sg13	DY344184	(ACA)10	14	0.01-0.36	271-312		
OBNJR4cn17	Contig2461	(AAAT)5	13	0.01-0.41	164-365

**Table 2:** Summary of allele distribution among clusters resulting from primary and secondary (nested) model-based cluster analyses

Cluster	Sample size	Total alleles	Average alleles per locus
k1	90	3081	1.75
k2	16	709	2.23
k3	36	907	1.34
Admixture	38	1812	2.58
k1.1	51	1760	1.76
k1.2	28	953	1.74
k2.1	8	363	2.27
k2.2	7	301	2.18
k3.1	7	189	1.41
k3.2	18	452	1.33
k3.3	11	266	1.30
Admixture	50	2225	2.49
Overall	180	6509	1.86

### Population structure and allele frequency

**Fig. 1:**
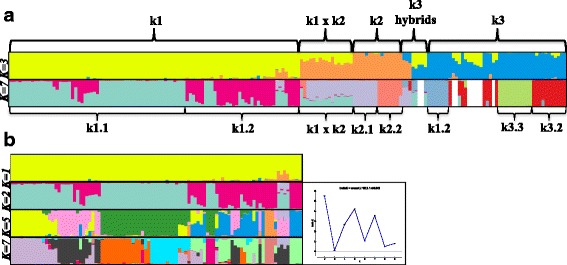
Primary and nested population structure for 180-accession panel of *Ocimum* species. Primary and secondary (nested) model-based clustering analysis using Structure ver 2.2.3 software for panels of *Ocimum* spp. accessions using 20 EST-SSR markers. **a** Major clusters (*K* = 3) (top histogram) and sub-clusters (*K* = 7) (bottom histogram) derived from primary and nested clustering iterations. Ten accessions were admixed and nested population structure could not be inferred due to unknown parentage of admixed primary cluster membership (white bars). **b** Major clusters (K = 1) (top histogram) and sub-clusters (*K* = 2,5,7) (bottom three histograms). ΔK statistic values for K = 2-10 (right). Accessions are ordered according to the unweighted pair group method using arithmetic average (UPGMA) clustering

Primary model-based clustering (population structure) analysis provided unambiguous evidence (Additional file 3) for three major clusters k1 (*n* = 90), k2 (*n* = 16) and k3 (*n* = 36) (Fig. 1a). The remaining 38 accessions were considered admixed due to *qI* < 0.70 and/or genotypes of bi-parental origin from different clusters (Additional file 1). The average number of alleles per locus among clusters differed with values of 1.75, 2.23 and 1.34 corresponding to k1, k2 and k3, respectively (Table 2). Furthermore, clear differences were observed in the distribution of alleles per locus among clusters derived from primary population structure analysis. The majority of loci in the k1 cluster had 2 alleles (57%), nearly twice the 33% observed for single-allele loci. A more even distribution was observed in the k2 cluster with 22%, 37% and 36% for one, two and three alleles per locus. This rate of tri-allelic loci is in stark contrast to the < 1% in k1 and k3 clusters and suggests three homozygous, homeologous loci among a proportion of markers in k2 cluster. A majority of loci in k3 cluster were mono-allelic, twice that of the 30% bi-allelic loci. Finally, the number of alleles for loci of admixed accessions was relatively evenly distributed from 1 to 4 ranging from 18% to 32%. Five alleles were observed in an additional 4.3% of admixed loci. A total of 1812 alleles and an average of 2.58 alleles per locus (highest among all clusters) were detected for the 38 admixed accessions indicating heterozygosity.

Secondary (nested) population structure analysis provided evidence for seven clusters (*K* = 7) among 142 non-admixed accessions (Fig. 1a). Two (*K* = 2) and three (*K* = 3) sub-clusters within k2 (k2.1 and k2.2) and k3 (k3.1, k3.2 and k3.3.), respectively, were strongly supported by the ΔK statistic (Additional file 4). Greatest support was provided for two (*K* = 2) sub-clusters, k1.1 and k1.2, among the 90-accession k1 primary cluster containing commercial sweet basil accessions. However, evidence was also provided for five (*K* = 5) and seven (*K* = 7) sub-clusters (Fig. 1b), suggesting cryptic population structure extant within this most economically important grouping. Nested population structure could not be determined for 9 accessions with admixed cluster membership from k1 and k3. Among these accessions, *qI* for either cluster (k1 or k3) failed to exceed 0.7 and could not be assigned due to unknown parentage. An additional 12 accessions had *qI* < 0.70 (Additional file 1) after being subjected to the nested population structure analysis and were considered admixed.

The average (Table 2) and distribution of the alleles per locus for sub-clusters was similar to the major primary clusters of k1 and k2. The range of alleles per locus among k1, k2 and k3 sub-clusters were 1.74-1.76, 2.18-2.27 and 1.30-1.41, respectively, indicating a high level of consistency with regard to allelic diversity. Interestingly, a relatively high percentage (11.2%) of null alleles occurred among accessions of sub-cluster k3.2.

### Molecular variance

Analysis of molecular variance (AMOVA) was performed using 142 and 130 non-admixed accessions determined by primary and nested model-based cluster analysis, respectively. Inter-population differentiation was supported at the *P* < 0.001 level for primary and nested population structure with Φ_PT_ values of 0.433 and 0.344, respectively (Table 3). The molecular variance due to differences within populations for nested clustering was 66% as compared to 57% from primary clustering. All pairwise Φ_PT_ values for (*K* = 3) clusters were significantly different (*P* < 0.001) (Table 4). The majority of pairwise Φ_PT_ values were highly significant (*P* < 0.001) for the seven (*K* = 7) clusters derived from nested analysis (Table 5). However, k3.1, k3.2 and k3.3 failed to demonstrate evidence for population differentiation based on AMOVA results (Table 5). These three sub-clusters contributed 51% of the total within population sum of squares (SS_WP_) while representing only 5.4% of the accessions included in the AMOVA (data not shown). Thus, high within population genetic variation coupled with a lower sample size may have lowered resolution for differentiation of these sub-clusters.

**Table 3:** Analyses of molecular variance (AMOVA) for non-admixed panel of *Ocimum* spp. using 20 EST-SSR markers among clusters resulting from primary and secondary (nested) model-based cluster analyses

Clustering Iteration	Source	df^a^	Sum of squares	Mean square	Estimated σ^2^	Molecular σ^2^
Primary^b^	Among^d^	2	997.3	498.6	13.0	43%
	Within^e^	139	2367.4	17.0	17.0	57%
	Total	141	3364.7		30.0	100%
Nested^c^	Among	6	996.9	166.1	9.0	34%
	Within	123	2108.7	17.1	17.1	66%
	Total	129	3105.7		26.1	100%

^a^Degrees of freedom

^b^Primary model-based clustering iteration (*K = 3*). Φ_PT_ = 0.433; *p* < 0.001

^c^Nested model-based clustering iteration (*K = 7*). Φ_PT_ = 0.344; *p* < 0.001

^d^ Among clusters

^e^Within clusters

**Table 4:** Pairwise Φ_PT_ estimates for clusters resulting from the primary model-based cluster analyses

Cluster	k1	k2	k3
k1	0.000	–	–
k2	0.576	0.000	–
k3	0.392	0.356	0.000

All Φ_PT_ estimates were highly significant (*p* < 0.001)

**Table 5:** Pairwise Φ_PT_ estimates for resulting from the secondary model-based cluster analyses

Sub-Cluster	k1.1	k1.2	k2.1	k2.2	k3.1	k3.2	k3.3
k1.1	0.000	–	–	–	–	–	–
k1.2	0.057	0.000	–	–	–	–	–
k2.1	0.572	0.600	0.000	–	–	–	–
k2.2	0.539	0.560	0.042^NS^	0.000	–	–	–
k3.1	0.376	0.389	0.354	0.243^*^	0.000	–	–
k3.2	0.418	0.406	0.361	0.289	0.000^NS^	0.000	–
k3.3	0.433	0.420	0.339	0.276	0.034^NS^	0.021^NS^	0.000

^NS^not significantly different *p* > 0.05, ^*^p < 0.05; all other Φ_PT_ estimates were highly significant (*p* < 0.001)

### Characterization of genetic relationships

**Fig. 2:**
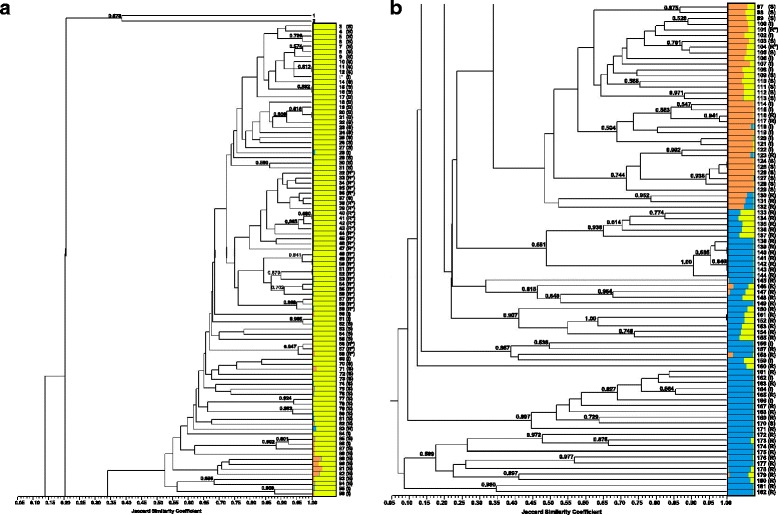
Primary population structure and UPGMA clustering for 180-accession panel of *Ocimum* species. Unweighted pair group method using arithmetic average (UPGMA) dendrogram aligned with primary model-based clustering of *Ocimum* spp. 180-accession panel and 2 outgroup accessions (1 and 2) using 20 EST-SSR markers. Genetic distance was calculated using the Jaccard Similarity Coefficient (x-axis) and bootstrap support values are the result of 1000 permutations with support value greater than 0.500 shown. Three clusters (*K*) were inferred using Structure ver 2.2.3 and membership to each cluster is represented by proportion of yellow, orange or blue colors within horizontal lines corresponding to each accession. Numbers right of membership histograms correspond to accessions. Response to downy mildew is indicated resistant (R) = DS < 1.0, intermediate (I) = 1.0 < DS < 3.0 or susceptible (S) = DS > 3.0. Accessions with response R* share a single source of resistance conferred by accession MRI. The figure is divided into two segments **a** (left) and **b** (right)

A cophenetic correlation value of *r* = 0.934 indicated strong goodness of fit [46] between the genetic similarity matrix used and the UPGMA dendrogram generated. Furthermore, the UPGMA dendrogram is in agreement with the placement of accessions for all major clusters (*K* = 3) determined by population structure analysis (Fig. 2a-b). The k1 cluster demonstrated generally lower genetic diversity as evidenced by high Jaccard similarity coefficient values (Fig. 2a). Importantly, accessions within the k1 cluster largely grouped consistent with known pedigree information available for 61% of accessions. The k2 cluster divides, according to nested population structure, into two clades of comparable genetic diversity. Although delineation of k3 into three sub-clusters was not supported by AMOVA, high bootstrap support values provide evidence of distinct groupings with high statistical backing (Fig. 2b). UPGMA grouping among k1 accessions did not demonstrate the same level of bootstrap support, however, this was not unexpected considering the ambiguity of nested model-based cluster analysis results.

The k1 cluster roots to a single major node that can be further divided into three distinct groups (Fig. 2a). This k1 cluster includes a commercial sweet basil clade that begins with ‘Caesar’ (Accession 3) and ends with ‘Lettuce Leaf Heirloom’ (Accession 31). Included are all accessions classified as *O. basilicum* such as the commercially important variety ‘Nufar’ (acc. 19) originally developed for FOB resistance and widely cultivated worldwide. In addition, all chilling (acc. 8-16) and FOB (acc. 13, 20-23, 27) tolerant Rutgers breeding lines are included in this group (Fig. 2a). A second major k1 clade includes two lineages RUMS (acc. 33-39) and RU4S (acc. 40-46, 48-59) (Fig. 2a) derived from breeding for DM_MRI_ resistance. The resistant genotypeMRI (acc. 47) is centrally located among accessions in these lineages. USDA-GRIN accession PI 197442 (acc. 60) and all remaining k1 accessions (Fig. 2a) vary substantially in phenotype and aromatic volatile composition (data not published). Accessions in this k1 clade are primarily cultivated for specialty or ornamental markets with the exception of ‘Eleonora’ (acc. 69), a variety marketed for commercial fresh market consumption.

A k1 x k2 admixed, hybrid clade is supported by UPGMA analysis and can be observed at the top of Fig. 2b. This clade includes F_1_, F_2_ or F_2:3_ progeny with low or no fertility from a k1 x k2  bi-parental cross. PI 172996 (acc. 116) and ‘Sweet Dani’ (acc. 115) comprise all k2 parents with the exception of RU_SB22XLIME_F1 (acc. 97) and PI_652060XRU_SB17_F1 (acc. 98).

The k2 cluster corresponds to a major clade, which is further divided into two groups (Fig. 2b). Nested population structure analysis (Fig. 1a) and bootstrap support values (0.504 and 0.744) (Fig. 2b) are both in agreement with this sub-division. Accessions in this cluster are described by their sources as *O. basilicum*, *O. citriodorum* or *O. americanum* (Additional file 1). Three accessions cluster separately from all other k2 accessions and constitute a k2 x k3 hybrid grouping. Another k1 x k3 admixed clade is well-supported (0.938) and includes three F_1_ hybrids derived from a cross between DM resistant k3 parent ‘Spice’ (acc. 139) and three different k1 accessions. Two additional USDA-GRIN accessions, PI 414201 (acc. 140) and PI 414203 (acc. 141), are included in this clade and parentage is unknown.

Although the division of k3 into sub-clusters k3.1, k3.2 and k3.3 suggested by nested population structure analysis was not supported by the pairwise Φ_PT_ estimates (Table 5), UPGMA provides evidence of clear delineations within this major cluster (Fig. 2b). The k3.1 cluster includes 7 phenotypically indistinguishable accessions sourced from commercial seed companies and the USDA-GRIN. This cluster is highly supported (1.00) and evidently an autonomous population (Fig. 1a; Fig. 2b). Another well-supported clade (0.997) is the k3.2 cluster (Fig. 1a; Fig. 2b), which includes 11 accessions beginning with PI 500952 (acc. 175) through PI 500942 (acc. 171). UPGMA analysis suggests this cluster represents a rather substantial juncture from the rest of the panel as it and subsequent clades are considered more basal than even the two *Nepeta cataria* outgroup accessions CN3 and CR9 according to UPGMA clustering. The k3.2 cluster is phenotypically homogenous and labeled *O. x africanum* according to the USDA-GRIN system. The k3.3 cluster includes basal most clades of the UPGMA dendrogram and the highest levels of genetic diversity based on Jaccard Similarity Coefficient genetic distance estimates shown as branch lengths in Fig. 2b. All accessions within this clade, supported by a 0.509 bootstrap value, are classified *O. gratissimum* according to their sources (Additional file 1). Within this clade bootstrap support values of 0.875, 0.977 and 0.897 support grouping of three more closely related pairs of accessions (Fig. 2b). PI 511865 (acc. 182) is labeled *O. selloi* and groups (0.980) with the phenotypically similar ‘Green Pepper Basil’ (acc. 181) (Fig. 2b). *O. gratissimum* and *O. tenuiflorum* accessions were used as outgroups in a previous AFLP-based assessment of basil phylogenetics [12], but did not include *O. selloi* found to be more basal in this study.

A number of additional accessions have membership to the k3.3 sub-cluster yet are more distally located from the basal end of the dendrogram (Fig. 1a). This includes phenotypically similar PI 652052 (acc. 145) and ‘Camphor’ (acc. 149); the former is classified *O. x africanum* by the USDA-GRIN, but more likely *O. kilimandsharicum* based on morphology and as described by Ben-Naim et al. [41]. These accessions group within a clade supported by a bootstrap support value of 0.907 (Fig. 2). A second, well-supported (0.867) clade included those with inferred ancestry to k3.3 (Fig. 1a). These accessions are classified *O. tenuiflorum* by commercial and USDA-GRIN sources (Additional file 1). Strong bootstrap support and phenotypic homogeneity among these two sets of accessions indicate UPGMA best resolves these k3 relationships.

### Geographic distribution among clusters

In contrast to the greater panel, information regarding origin is available for the majority of k3 accessions (Additional file 1). All accessions in the k3.2 cluster were collected in Zambia indicating this grouping may be native to south-central Africa. Accessions in k3.1 and k3.3 are far less geographically congruent. Sub-cluster k3.3 includes diverse New and Old World geographic collections ranging from South America (PI 511865; Uruguay) to South East Asia (PI 652055; Sri Lanka) to Sub-Saharan Africa (PI 652064; Tanzania). The collection locations of four known accessions in k2.1 are split between Turkey (PI 172996; PI 172998) and Iran (PI 253157; 296391) suggesting a Middle-Eastern grouping. The remaining k2.1 accessions are cultivars with unknown parentage developed for a volatile composition high in monoterpenoids geranial and neral (i.e. citral). The k2.2 sub-cluster includes PI 652060 and PI 652061 collected in Pakistan and India, respectively, however, geographic origin of the remaining five accessions are unknown. The k1 cluster is comprised of largely commercial and Rutgers breeding lines in which parentage or geographical origin of parents is unknown. Ten of 12 USDA-GRIN accessions were collected in Turkey, Iran or Macedonia.

### Distribution of DM resistance

Two unique sources of DM resistance (mean DS < 1.0), MRI (acc. 47) and Kivumbisi Lime x RUSB_17 F1 (acc. 83), were identified in the major cluster k1. The former accession has been used to develop DM_MRI_ resistant breeding lines, while the latter is a sterile F_1_ hybrid with DM resistance conferred from the admixed accession 153 ('Kivumbisi Lime'). Apart from these two sources of resistance, the k1 cluster was largely DM susceptible with a mean DS of 3.6. Seven accessions demonstrated an intermediate response to DM (1.0 < DS < 3.0) and may be incorporated into breeding strategies targeting durable resistance [26].

**Fig. 3:**
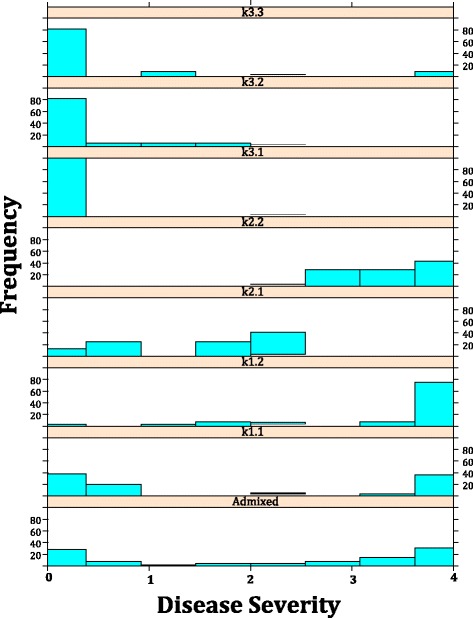
Downy mildew response phenotype distribution among *Ocimum* species clusters. Distribution of disease severity among sub-clusters derived from secondary (nested) model-based clustering analysis using Structure ver 2.2.3 software

The frequency of DM intermediate and resistant accessions increased with genetic distance from the k1 cluster. Intermediate resistance was observed among the majority of k2 accessions reflected by the 2.27 mean DS for this cluster. Interestingly, the k2.1 mean DS of 1.5 was lower than for k2.2 (mean DS = 3.4) for which the lowest DS was 2.9 (Fig. 3). Major cluster k3 is the primary source of DM resistance (mean DS = 0.36) with 29 resistant, 4 intermediate and one susceptible genotype. The highly supported (bootstrap = 0.867) clade of four accessions 156 - 159 exhibited hypersensitive response (HR). This response was not completely effective in preventing sporulation for these accessions such as 159 (PI 652057) (DS = 2.57) (Additional file 1).

Fourteen F_1_ hybrid accessions developed from DM resistant parents demonstrated resistance with the exception of SB17 x 172996 F1 (acc.100) with DS = 2.75. These results are in agreement with previous evaluation of F_1_ progeny providing corroborating evidence of dominant gene action [41]. Three k1 x k3 DM resistant F_1_ hybrids developed from ‘Spice’ (acc.133), ‘Kivumbisi Lime’ (acc. 153) and ‘Camphor’ (acc. 149) exhibit introgressed k3 resistance but were completely sterile. Hybrids from k1 accessions crossed with DM resistant and intermediate k2.1 accessions PI 172996 (acc. 116) and ‘Sweet Dani’ (acc. 115) demonstrated low fertility that could be overcome, however, an intermediate and susceptible response was observed for the respective F1 progeny (Additional file 1). Thus, unlike k3 accessions resistance did not appear to be under dominant gene control and requires further investigation to characterize the mechanism of DM response.

## Discussion

### Population structure and genetic diversity

Model-based and UPGMA clustering analyses provide evidence of three distinct delineations and 15.6% admixture among the 180 *Ocimum* spp. accession panel. Nested population structure analysis provides additional evidence for seven sub-populations. Greatest support (Δ*K*) was provided for two distinct populations (*K* = 2) nested within the most economically important *O. basilicum* k1 cluster. However, the possibility of five and seven sub-populations could not be discounted (Fig. 1b). Cryptic population structure in the k1 cluster may be attributed to extensive interbreeding, which is particularly prevalent in the *O. basilicum* species [47]. High levels of admixture were also observed for the K = 5 and K = 7 population models (Fig. 1b), further supporting the exchange of genomic content potentially as a function of natural and purposeful outcrossing. This is most evident in the k1.2 cluster, where both admixture (Fig. 1b) and genetic distance increase (Fig. 2a) proceeding towards the basal end of the dendrogram containing a large proportion of cultivars and USDA-GRIN accessions (Additional file 1). These results indicate plant breeding and natural outcrossing have maintained some level of genetic diversity within this economically salient cluster of accessions.

The k1 cluster was the largest in this study with 90 accessions, yet best evidence was provided for only two sub-clusters, which was also the case for the 16-accessions in k2. Furthermore, the 36-accession k3 cluster resolved into three sub-clusters and may potentially contain additional populations based on interpretation of UPGMA analysis (Fig. 2b). While these results do not necessarily suggest a bottleneck in k1, the k2 and k3 clusters clearly exhibit far greater genetic diversity. Introgression of accessions from the k2 and k3 clusters would be advantageous in broadening the k1 (*O. basilicum*) gene pool.

### Taxonomical discrepancies

Results of this study find genetic distance and population inference is often not correlated with reported accession collection location, phenotype or even species epithet. Conflicting and redundant nomenclature in the literature, the USDA-GRIN system and commercial seed sources have confounded accurate species-level assignment to important *Ocimum* spp. accessions [24]. Phenotype appears to be somewhat predictive of genotype in clusters k3.1 and k3.2, however, leaf shape, habit, flower morphology and volatile composition are clearly heterogeneous among the remaining sub-clusters. For instance, k2.1 accessions 115 ('Sweet Dani') and 116 (PI 172996) group within a single clade (0.553), however, Sweet Dani exhibits no anthocyanins and a volatile composition that is 68% citral [8], while PI 172996 exhibits leaf and stem anthocyanins and a volatile composition 91% methyl chavicol [USDA-GRIN]. Meanwhile, k1.2 cluster accesions 77 ('Queenette') and 78 ('Sweet Thai') contain stem anthocyanins and high methyl chavicol comparable to PI 172996. Although phenotype-based classification may provide some predictive measure of genetic relatedness, applications such as plant breeding require highly accurate and precise understanding of relations among accessions in order to construct effective selection strategies.

### Ploidy and reproductive barriers

Interesting differences in the mean and distribution of alleles per locus were observed among major and sub-clusters in this study. The majority of non-admixed accessions included in the panel are considered highly inbred and therefore homozygous across loci. Given this general assumption, some inferences can be made with regard to ploidy. A majority bi-allelic loci among k1 accessions suggests this cluster is an allotetraploid with two homozygous loci representing two sub-genomes. This system has been previously observed for the well-characterized allotetraploid genomes of *Brassica napus* [32] and *Gossypium hirsutum* [48]. One and two alleles per locus were observed in 33 and 57% of loci in the k1 cluster, respectively. These results are similar to the distribution of alleles per locus in a 240 EST-SSR marker survey using inbred k1 genotypes SB22 and MRI, in which a polymorphic subset also demonstrated disomic inheritance [17].

Among accessions in the k2 cluster, a 36% rate of three alleles per locus provides evidence of three homeologous loci, suggesting an allohexaploid genome. Previous investigations of cytology and nuclei acid content for the species *O. africanum* (syn. *O. citriodorum*) [20] suggested an allohexaploid (2n = 6× = 72) genome structure [11, 13, 18, 19]. Allelic distribution and cluster analysis in this study both support the hypothesis of an allohexaploid *O. africanum* k2 cluster and a need for nomenclature to be standardized across these accessions. Confusingly, USDA-GRIN accessions in the strongly supported (0.997) sub-cluster (k3.2) are labeled as *O. xafricanum*, illustrating the continued *Ocimum* spp. taxonomic issues and a need for revision of nomenclature. Hybridization of k1 and k2 accessions was previously characterized as an interspecific crosses with low fertility [11, 19]. All 17 k1 x k2 F_1_, F_2_ and F_2:3_ accessions in this study demonstrated low fertility. This is in agreement with a previous report of low fertility of the PI 172996 (acc. 116) x ‘Peri’ F_1_ hybrid [41]. In this study *O. basilicum* accession ‘Peri’ is replaced with SB17 (acc. 20) and used as recurrent parent to generate the RU172S BC_1_ progeny (Fig. 2a). Three RU172S BC_1_ individuals demonstrated increased fertility relative to the F_1_, suggesting restoration of reproductive viability may be correlated with increased k1 membership and providing a platform for development of near isogenic lines (NILs). Thus, introgression of traits such as disease resistance from k2 accessions to commercial sweet basil is possible through backcross breeding.

A majority (60%) of single allele loci among k3 accessions was nearly two- and three-fold greater than k1 and k2, respectively. One of two scenarios are likely to explain the predominance of single-allele EST-SSRs among k3 accessions. First, it is possible that a divergent sub-genome is shared among k3 accessions, but does not have adequate homology for transferability of SSR markers derived from *O. basilicum* EST database. This would suggest a system similar to triplicated *Brassica* genomes in which *B. napus* AACC and *B. juncea* AABB have only the A genomes in common [49]. A second possibility is the complete absence of a sub-genome, which would suggest k3 as a diploid cluster. A diploid genome hypothesis was previously proposed for k3 *O. gratissimum* and *O. tenuiforum* accessions [11, 15], which have consistently lower 2C-values [12, 13]. Furthermore, the recently sequenced genome of *O. tenuiflorum* genotype ‘CIM Ayu’ is described as a diploid (2n = 2× = 16) with a 386 Mbp genome [15], more than ten-fold less than tetraploid *O. basilicum* based on 2C estimates [12, 13]. Koroch et al. [13] reported a 2C-value for k3.3 accession 160 (PI 652066, *O. campechianum*) significantly lower (*p* < 0.001) than k3.1 accession 143 (PI 652059, *O. tenuiflorum*). Accession 182 (PI 511865) was the most basal accession in this study (Fig. 2b), yet is reported to have a 2C-value comparable to PI 652059 (acc. 143) [13]. Thus, comparison of previously reported nucleic acid content with allelic distribution, genetic distance estimates and cluster analyses in this study suggest a complex *Ocimum* spp. genome evolution with multiple chromosomal accumulation and/or deletion events.

Three F_1_ progeny of k3.1 accession 139 ('Spice') hybridized with *O. basilicum* k1.1 accessions 22 (RUSB_09), 6 ('DiGenova') and 47 (MRI) form a well-supported (0.938) clade with admixed accessions 135 (PI 414201) and 136 (PI 414203) (Fig. 2b). Sterility among these progeny suggest a major reproductive barrier between commercially important *O. basilicum* k1.1 accessions and highly supported (1.00) k3 clade. Ben-Naim et al. reported F_1_ sterility among progeny of DM resistant accessions 161 (PI 500945) and 168 (PI 500950) hybridized with *O. basilicum* accession ‘Peri’ [41]. These accessions are found in the k2.2 sub-cluster suggesting the k1 x k3 F1 sterility barrier extends to this more basal clade (Fig. 2b). Surprising k1 *qI* among a number of non-hybrid k3 accessions 149-155 (Fig. 2b) suggests the possibility of recently shared ancestry between these clusters. However, sterility among Kivumbisi Lime x SB17 progeny demonstrates high k1.1 *qI* does not correspond to fertility. Viable F_1_ progeny could not be obtained from cross-pollination of k1.1 and k3.3 accessions. This is consistent with at least one previous report of attempted *O. basilicum* x *O. tenuiflorum* hybridization [47].

Although evidence of varying ploidy levels is a suspected cause of reproductive barriers among wide crosses [41] the exact mechanism remains unclear. Comparative genomic analysis of cultivated and wild *Solanum* spp. demonstrate seed and pollen infertility can be controlled by a small number of loci with shared evolutionary history [50]. Further investigation is needed to determine the cause of infertility among *Ocimum* spp.

### Distribution of DM resistance and breeding

Forty-three unique sources of genetic resistance to DM are identified from greenhouse screening in this study. These resistant  accessions are heavily concentrated in the k3 cluster with 29 from 7 species according to the sources from which they were obtained (Additional file 1). An additional 9 resistant accessions had majority k3 *qI* membership and UPGMA placement among k3 accessions (Fig. 2b). 116 (PI 172996), 117 (PI 172998) and 119 (PI 296391) were first identified as having little or no disease incidence by Pyne et al. 2013. These accessions demonstrated minimal leaf sporulation in this study (DS ≤ 0.63) and were determined to be in a population (k2) distinct from commercial sweet basil (k1). MRI (acc. 47) represents the only source of DM resistance (DM_MRI_) identified in the *O. basilicum* k1 cluster. Recently discovered QTL *dm11.1* demonstrated dominant gene action conferred by MRI (acc.47), but detection of additional minor QTL explained additional phenotypic variance and a somewhat complex mechanism for highest levels of resistance [17].

These accessions represent candidates for field confirmation of resistance to *P. belbahrii* and, ultimately, DM resistance breeding. Ben-Naim et al. found DM response in greenhouse and field experiments to be largely correlated. Response of accessions common across this and previous greenhouse studies [41, 42] is generally consistent with minor differences. Differential disease response of genotypes across studies may be due to a number of confounding effects including environment, inoculum concentration and rating system [51]. Another possibility of greater consequence to disease control is the potential occurrence of pathogen evolution resulting in races exhibiting differential host virulence. Proliferation of races is common among the most economically devastating downy mildews such as *Pseudoperonospora cubensis* [52], *Bremia lactuca* [37] and *Hyaloperonospora brassicae* [53]. Phylogenetic characterization of *P. belbahrii* isolates is needed to determine the range of genetic diversity and identify possible extant pathogen races.

Candidacy of accessions for DM resistance breeding is contingent upon sexual compatibility and reproductive capacity with commercial sweet basil found in sub-cluster k1.1 (Fig. 2a). DM resistance from k1.1 accession 47 (MRI) is the only documented characterization [17, 54] and introgression of genetic resistance beyond F_1_ progeny. Partial sterility observed for k1 x k2 crosses appears to be surmountable by backcrossing as demonstrated by the ‘RU172S17’ BC_1_ accessions 89-92 (Fig. 2a). This represents a potential strategy for introduction of k2 genomic DNA associated with important traits such as intermediate DM resistance (Table 1) and FOB resistance available in many of these accessions. An intermediate DM response (DS = 2.75) for the SB17 x PI 172996 F1 hybrid was significantly (*P* < 0.05) higher than resistant parent PI 172996 and closer to the mid-parent DS of 2.18 suggesting resistance may be quantitative. The heritability of DM resistance from k2 accessions such as PI 172996 is unclear and requires further investigation.

The k3 cluster represents a rich source of genetic resistance. Furthermore, consistent non-significant differences in disease response for F_1_ progeny and their resistant parent indicate dominant gene action is widespread among these candidates [41]. Qualitative and, in particular, single dominant gene control of DM is well documented across plant species [37, 55, 56]. HR in *O. tenuiflorum* accessions 156-159 indicates effector triggered immunity [57], a response distinct from the remaining k3 accessions in which no signs or symptoms were observed. It remains unclear whether these accessions have evolved unique resistance genes, but nonhost resistance is more likely among distant relatives of susceptible k1 accessions. Introgression of nonhost resistance from wild lettuce relatives *Lactuca serriola* and *Lactuca saligna* to commercial *L. sativa* is being used to build durable DM resistance [37, 58]. Further investigation is needed to identify redundancy of resistant genes, especially among closely related accessions. 

## Conclusions

Results of this study provide a robust characterization of population structure, genetic diversity and response to DM among *Ocimum* spp. EST-SSRs provide a useful tool for increased resolution of this genus through continued germplasm collection and genotyping. Although evidence is concurrent for allotetraploid and allohexaploid genomes among economically important k1 and k2 clusters, respectively, the highly diverse and DM resistance-rich k3 cluster remains complicated by discrepancies in reports of genome size. Characterization of subgenomes as shared or distinct among allopolyploid species and identification of a diploid progenitor(s) are needed to elucidate genome structure.

As breeding among specialty crops becomes increasingly prevalent, more sophisticated tools will be required. This trend is evidenced in *Ocimum* spp. by recent development of a first draft genome [15], de novo meta-transcriptomics [59, 60] and genetic/QTL mapping [17]. Determination of major and cryptic population structure as well as phylogenetic classification among DM resistant candidates provides another important resource for accelerated genetic improvement.

## Methods

### Plant material

A 180-accession panel of *Ocimum* spp. was selected to provide a genus-wide representation of genetic diversity, while targeting germplasm developed primarily for improved performance in important agronomic traits at Rutgers University (Additional file 1). Eight chilling (“RUCB”) and 7 *Fusarium oxysporum* f.sp. *basilici* (FOB) (“RUSB”) tolerant *O. basilicum* inbred lines were selected. Twenty-nine breeding lines were included from two lineages (“RU4S” and “RUMS”) selected for DM resistance. Eighteen F_1_ hybrids were developed from hybridization of 9 *Ocimum* spp. accessions of varying DM response with 4 DM-susceptible *O. basilicum* inbred lines. All cross-pollinations were performed according to Pyne et al. [54]. Seven F_2_ selections and a single F_2:3_ individual were included. Four first-generation backcross lines selected for FOB resistance (“RU172S”) were also included. Remaining accessions were obtained from either commercial seed companies or the USDA-GRIN repository (Aimes, IA), ultimately representing 10 species according to these sources. Finally, two catnip (*Nepeta cataria*) accessions (CN3 and CR9) were included as outgroup species.

### Disease rating (phenotypic evaluation)

Seed from commercial, USDA-GRIN and inbred accessions were planted in Fafard Growing Mix 2 (Sun Gro Horticulture, Agawam, MA) and germinated under intermittent misting. Hybrid and non-flowering accessions (‘Pesto Perpetuo’, ‘GCB’ and ‘Pezou’) were cloned by vegetative cutting from mother plants under intermittent misting. The *P. belbahrii* isolate collected at the Rutgers Agricultural Research and Extension Center (RAREC) in 2013 and maintained on susceptible check variety ‘DiGenova’ was used in this study. Inoculum was prepared and applied to first true leaf sets according to Pyne et al. [61]. Eight seedlings (individuals) per each accession were inoculated and disease ratings were performed 10 days post inoculation (DPI) corresponding to the interval at which disease severity plateaus under these greenhouse conditions. Individual plants were assigned a disease severity score using an ordered categorical scale in which 0 = no sporulation, 1 = 1-10%, 2 = 11-25%, 3 = 26-50%, 4 = 51-100% [54]. A disease severity score (DS) was assigned to each accession from the average of 8 individuals for two, repeated experiments.

### EST-SSR genotyping

Young leaves were harvested from axillary nodes of each accession in this study, frozen and then ground using mortar and pestle. Genomic DNA (gDNA) was extracted from ~ 80 mg of ground leaf tissue for all accessions using the E.N.Z.A. SP Plant DNA Kit (Omega BioTek, Norcross, GA).

A set of 240 EST-SSR markers were previously developed and used to genotype the accessions MRI and SB22 [17]. Preliminary experiments identified 20 di-, tri- and tetranucleotide repeat markers demonstrating reliable, unambiguous amplification across replicated PCR evaluations in a diverse subset of *Ocimum* spp. accessions. Given the putative allopolyploid genome structure of these species, markers were considered to be amplified in shared sub-genomes and, thus, orthologous. Seven of the 20 selected markers were previously mapped to a sweet basil linkage map developed from MRI x SB22 F_2_ mapping population [17]. All primers were synthesized by Integrated DNA technologies (Coralville, IA). Multiplexed PCR was performed using conditions and fluorescent dyes (FAM, NED, PET and VIC) previously described [17]. Fragments generated from PCR were separated by capillary electrophoresis using an ABI 3500xL Genetic Analyzer (Life Technologies Corporation, Carlsbad, CA). Genemapper 4.1 (Applied Biosystems) software was used to determine fragment size for alleles across all accessions and exported for downstream analysis.

Polyploidy is a confounding factor in population genetic studies due to the complication of allelic configuration among genotypes. In allopolyploid accessions of a single ploidy amplification of loci in independent sub-genomes provides a platform for conversion to a diploid SSR genotyping system [48, 62]. Accurate, multi-allelic (codominant) genotype assignment of complex germplasm panels with multiple ploidy levels in the absence of subgenome assignment exceeds current data analysis capabilities. Genotype data in this study was therefore scored as binary (presence = 1; absence/null = 0) as suggested by Honig et al. [63]. SSR markers absent of any alleles (primer site mutation) or having many (> 6) alleles (non-specific primer binding) were considered null and coded ‘0’. Genotypes were coded ‘-1’ in the case of missing data. Polymorphic information content (PIC) was calculated for individual alleles using the formula 2P_i_Q_i_, where P_i_ is the frequency of presence and Q_i_ is the frequency of absence for a given allele [63]. The binary genotype matrix for all accessions and SSR markers used in this study can be found in Additional file 5.

### Phenotype data analysis

Analysis of variance (ANOVA) was performed using R software to determine whether the effect of genotype was significant with respect to the response variable, DS. The agricolae package in R was used to perform DS mean separation using Tukey’s honest significant difference (HSD) test with α = 0.05. All accessions were included in this analysis with the exception of PI 511865, which was found to segregate and, thus, could not be treated as a single genotype.

### Population structure analysis

To determine population structure, a nested Bayesian model-based clustering approach described by [64] was implemented with Structure ver 2.3.4 software [44] to infer assignment of individuals to *K* clusters and sub-clusters. This approach has been effective in identifying important major and cryptic population structure in various plant species including apple [65], cotton [48] and rice [66]. All simulations were performed using the ‘admixture model’ with ‘correlated allele frequencies’. The algorithm was executed with parameters set to a burnin of 100,000 followed by 200,000 Markov Chain Monte Carlo (MCMC) repetitions with 10 replicated runs per *K*. Initial (primary) Structure analysis was performed for *K* = 1 – 15 using all 180 accessions to determine major delineations in overall population structure. Evanno’s method [67] was employed to provide an estimate of the most likely number of clusters using Structure Harvester software v0.6.94 [68] to determine the natural logarithm probability Pr(X|K) and ad hoc ΔK statistic (mean(|L”(*K*)|) / sd(L(*K*)) from membership coefficient (*qI*) matrices. Following selection of the optimal *K* for all replicated runs, the FULLSEARCH algorithm of CLUMPP software [69] was used to identify the optimal permuted order of *qI* matrices with the greatest pairwise similarity. The average of this permutation provided the best *qI* matrix representation, which was visualized using DISTRUCT software [70].

Determinations of the *qI* threshold at which to infer cluster assignment vary by study, but range from *qI* = 0.5 [71] – 0.8 [65]. Inclusion of wide crosses (F_1_, F_2_ and BC_1_) in this study provided a measure of high admixture, which informed the selection of 0.70 as an appropriate *qI* threshold. Thus, accessions were assigned to the cluster for which *qI* > 0.70, while those with a maximum *qI* less than 0.70 were considered admixed. In addition, hybrid (F_1_, F_2_, F_2:3_) and first backcross (BC_1_) accessions with parentage assigned to two different clusters were considered admixed.

Nested (secondary) Structure analysis was performed separately for non-admixed accessions of each cluster ascertained from the primary analysis. Algorithm parameters were identical to that of the primary analysis but included *K* = 1-10. Hybrid and backcross accessions were then assigned to sub-clusters by replacing the primary *qI* with secondary *qI* determined for each parent. Accessions for which parentage was unknown were excluded from secondary Structure analysis. Assignment to sub-clusters was repeated using the same admixture criteria as in the primary analysis.

### AMOVA

The integrity of clusters derived from population structure was further investigated by performing an analysis of molecular variance (AMOVA) using GenAlEx 6.501 software [72]. A Nei [73] genetic distance matrix was first calculated using the binary genotype matrix previously described for the model-based clustering analyses. Two separate AMOVA were performed to partition within and among population estimated and molecular variance (σ^2^) components. Statistical significance of pairwise genetic distance among (i) primary and (ii) secondary clustering iterations were used to calculate the pairwise population PhiPT (Φ_PT_) test statistic, which provides a measure of interpopulation genetic diversity with intra-individual (heterozygosity) variation suppressed. The Φ_PT_ statistic was calculated for all pairwise cluster combinations, which were determined significantly different (*p* < 0.05) by 999 random permutations of the data.

### UPGMA

To investigate genetic relationships among accessions in the full panel, unweighted pair group method using arithmetic average (UPGMA) clustering was performed with Numerical Taxonomy System (NTSYSpc) ver 2.21q software (Exeter Software, Setauket, New York, USA) [46]. Genotype data from outgroup accessions CN3 and CR9 (*Nepata cataria*) were added to the same binary genotype matrix used for population structure and AMOVA analyses resulting in a total of 182 accessions. A genetic similarity matrix was generated using the Jaccard similarity coefficient method [74] in the NTSYSpc SIMQUAL module. Cluster analysis was then performed by UPGMA in the SAHN module and the output was visualized as a dendrogram with the TREE module. A Mantel test was performed with 999 test permutations using the MXCOMP module to determine goodness of fit between the genetic similarity matrix and the UPGMA dendrogram converted to cophenetic values with the COPH module. Finally, the original binary genotype matrix was resampled 1000 times using the RESAMPLE module and the results were used as input for the CONSENS module to calculate bootstrap values using the majority rule method and a minimum support value of 0.500.

## Additional files

Additional file 1: Description of 180-accession panel of *Ocimum* spp., cluster membership and response to downy mildew (*Peronospora belbahrii*) reported as disease severity. (PDF 115 kb)

Additional file 2: Primer sequences and melting temperatures (T_m_) for the EST-SSRs used in this study. (PDF 52 kb)

Additional file 3: Structure Harvester output for primary model-based clustering using Structure ver 2.3.4 software. Plot of Δ*K* (left) and LnP(D) (right) for *K* = 1 – 15. (PDF 126 kb)

Additional file 4: Structure Harvester output for secondary (nested) model-based clustering using Structure ver 2.3.4 software. Plots of Δ*K* (left) and LnP(D) (right) for *K* = 1 – 10. Three nested analyses shown for k1 (top), k2 (middle) and k3 (bottom) primary clusters. (PDF 215 kb)

Additional file 5: Genotype data. Binary matrix of accessions (rows) and alleles (columns). (XLSX 186 kb)

## Abbreviations

AM: Association mapping
AMOVA: Analysis of molecular variance
ANOVA: Analysis of variance
BC: Backcross generation
cDNA: Chloroplast DNA
cM: CentiMorgan
DM: Downy mildew
DM_MRI_: MRI-conferred downy mildew resistance
DPI: Days post inoculation
DS: Disease severity
EST: Expressed sequence tag (EST)
FOB: *Fusarium oxysporum* f.sp. *basilica*
gDNA: Genomic DNA
HR: Hypersensitive response
HSD: Tukey’s honest significant difference
ISSR: Inter-simple repeat markers
MCMC: Markov Chain Monte Carlo
NCBI: National Center for Biotechnology Information
NIL: Near isogenice lines
NTSYSpc: Numerical Taxonomy System
PIC: Polymorphism information content
SNP: Single nucleotide polymorphism
SSR: Simple sequence repeat
SS_WP_: Within population sum of squares
T_m_: Melting temperature
UPGMA: Unweighted pair group method using arithmetic average
USDA-GRIN: United States Department of Agriculture National Genetic Resources Program
