# Supplementary material for: Population structure, genetic diversity and downy mildew resistance among Ocimum species germplasm
Source: BMC Plant Biol. 2018 Apr 23;18:69. doi: 10.1186/s12870-018-1284-7 (PMC5914031; doi:10.1186/s12870-018-1284-7)
Supplement: Supplementary file 1 — Description of 180-accession panel of Ocimum spp., cluster membership and response to downy mildew (Peronospora belbahrii) reported as disease severity. (PDF 115 kb) [file 12870_2018_1284_MOESM1_ESM.pdf]

Additional file 1. Description of 180-accession panel of *Ocimum* spp., cluster membership and response to downy mildew (*P. belbahrii*) measured as disease severity (DS)

Entry ID	Origin/ Source[a]	Accession	Species[b]	Cluster[c]	Sub-Cluster	DS[e]	
3	HSC	Caesar	*O. basilicum*	k1	k1.1	4.00	a
4	Rutgers	RUSB_23	*O. basilicum*	k1	k1.1	3.75	abc
5	EZ	Genovese Martina	*O. basilicum*	k1	k1.1	4.00	a
6	SSC	DiGenova	*O. basilicum*	k1	k1.1	4.00	a
7	EZ	Eowyn	*O. basilicum*	k1	k1.1	4.00	a
8	Rutgers	RUCB_01	*O. basilicum*	k1	k1.1	3.88	ab
9	Rutgers	RUCB_39	*O. basilicum*	k1	k1.1	3.50	abcde
10	Rutgers	RUCB_04	*O. basilicum*	k1	k1.1	3.75	abc
11	Rutgers	RUCB_10	*O. basilicum*	k1	k1.1	4.00	a
12	Rutgers	RUCB_31	*O. basilicum*	k1	k1.1	4.00	a
13	Rutgers	RUSB_13	*O. basilicum*	k1	k1.1	2.38	fghij
14	Rutgers	RUCB_16	*O. basilicum*	k1	k1.1	3.13	abcdef
15	Rutgers	RUCB_14	*O. basilicum*	k1	k1.1	3.63	abcd
16	Rutgers	RUCB_19	*O. basilicum*	k1	k1.1	3.75	abc
17	Rutgers	Poppy Joes	*O. basilicum*	k1	k1.1	4.00	a
18	JSS	Italian Large Leaf	*O. basilicum*	k1	Admixed	4.00	a
19	JSS	Nufar	*O. basilicum*	k1	k1.1	4.00	a
20	Rutgers	RUSB_17	*O. basilicum*	k1	k1.1	3.63	abcd
21	Rutgers	RUSB_22	*O. basilicum*	k1	k1.1	4.00	a
22	Rutgers	RUSB_09	*O. basilicum*	k1	k1.1	4.00	a

23	Rutgers	RUSB_05	*O. basilicum*	k1	k1.2	3.88	ab
24	Greece	PI 263870	*O. basilicum*	k1	k1.1	3.88	ab
25	RSS	Marseilles	*O. basilicum*	k1	k1.2	3.63	abcd
26	Maryland	PI 414197	*O. basilicum*	k1	k1.2	4.00	a
27	Rutgers	RUSB_06	*O. basilicum*	k1	Admixed	4.00	a
28	Rutgers	RU_003_4	*O. basilicum x O. americanum*	k1	Admixed	2.75	defgh
29	Hawaii	Haw1	*O. basilicum*	k1	Admixed	4.00	a
30	JSS	Napoletano	*O. basilicum*	k1	Admixed	4.00	a
31	HSC	Lettuce Leaf Heirloom	*O. basilicum*	k1	Admixed	4.00	a
32	Rutgers	MRI x RUSB_22 F1	*O. basilicum*	k1	k1.1	0.88	nopqrs
33	Rutgers	RUMS_707	*O. basilicum*	k1	k1.1	0.75	opqrs
34	Rutgers	RUMS_707010103	*O. basilicum*	k1	k1.1	0.75	opqrs
35	Rutgers	RUMS_569	*O. basilicum*	k1	k1.1	0.38	qrs
36	Rutgers	RUMS_46911	*O. basilicum*	k1	k1.1	0.63	pqrs
37	Rutgers	RUMS_498	*O. basilicum*	k1	k1.1	4.00	a
38	Rutgers	RUMS_469	*O. basilicum*	k1	k1.1	0.56	qrs
39	Rutgers	RUMS_394	*O. basilicum*	k1	k1.1	0.63	pqrs
40	Rutgers	RU4S_33	*O. basilicum*	k1	k1.1	0.25	rs
41	Rutgers	RU4S_36	*O. basilicum*	k1	k1.1	0.13	rs
42	Rutgers	RU4S_37	*O. basilicum*	k1	k1.1	0.13	rs
43	Rutgers	RU4S_50	*O. basilicum*	k1	k1.1	0.38	qrs
44	Rutgers	RU4S_0177	*O. basilicum*	k1	k1.1	0.50	qrs

45	Rutgers	RUMS_394600115	*O. basilicum*	k1	k1.1	0.63	pqrs
46	Rutgers	RU4S_0741	*O. basilicum*	k1	k1.1	0.38	qrs
47	SMS	MRI	*O. basilicum*	k1	k1.1	0.25	rs
48	Rutgers	RU4S_07410401	*O. basilicum*	k1	k1.1	0.13	rs
49	Rutgers	RU4S_07410402	*O. basilicum*	k1	k1.1	0.13	rs
50	Rutgers	RU4S_07410403	*O. basilicum*	k1	k1.1	0.13	rs
51	Rutgers	RU4S_42210101	*O. basilicum*	k1	k1.1	0.25	rs
52	Rutgers	RU4S_42210102	*O. basilicum*	k1	k1.1	0.25	rs
53	Rutgers	RU4S_42210103	*O. basilicum*	k1	k1.1	0.25	rs
54	Rutgers	RU4S_26243301	*O. basilicum*	k1	k1.1	0.13	rs
55	Rutgers	RU4S_26243302	*O. basilicum*	k1	k1.1	0.13	rs
56	Rutgers	RU4S_26243303	*O. basilicum*	k1	k1.1	0.13	rs
57	Rutgers	RU4S_47082301	*O. basilicum*	k1	k1.1	0.75	opqrs
58	Rutgers	RU4S_47082302	*O. basilicum*	k1	k1.1	0.75	opqrs
59	Rutgers	RU4S_47082303	*O. basilicum*	k1	k1.1	0.75	opqrs
60	Ethiopia	PI 197442	*O. basilicum*	k1	k1.2	1.88	hijklm
61	RSS	Globette	*O. basilicum*	k1	Admixed	2.88	cdefg
62	RSS	Minette	*O. basilicum*	k1	Admixed	3.50	abcde
63	SOC	Cinnamon2	*O. basilicum*	k1	k1.2	4.00	a
64	SSC	Red Genovese	*O. basilicum*	k1	k1.2	3.63	abcd
65	Turkey	PI 170581	*O. basilicum*	k1	k1.2	4.00	a
66	Rutgers	RUMS_394601241101	*O. basilicum*	k1	k1.1	0.25	rs
67	Rutgers	RUMS_394601241102	*O. basilicum*	k1	k1.1	0.25	rs

68	Rutgers	RUMS_394601241103	*O. basilicum*	k1	k1.1	0.25	rs
69	EZ	Eleonora	*O. basilicum*	k1	Admixed	1.63	jklmno
70	Turkey	PI 175793	*O. basilicum*	k1	k1.2	4.00	a
71	Iran	PI 190100	*O. basilicum*	k1	k1.2	3.88	ab
72	Macedonia	PI 368697	*O. basilicum*	k1	k1.2	3.38	abcde
73	Macedonia	PI 358469	*O. basilicum*	k1	k1.2	4.00	a
74	RSS	Magical Michael	*O. basilicum*	k1	k1.2	4.00	a
75	Macedonia	PI 358464	*O. basilicum*	k1	k1.2	4.00	a
76	Macedonia	PI 368700	*O. basilicum*	k1	k1.2	4.00	a
77	RSS	Queenette	*O. basilicum*	k1	Admixed	4.00	a
78	JSS	Sweet Thai	*O. basilicum*	k1	k1.2	4.00	a
79	RSS	Thai Siam Queen	*O. basilicum*	k1	k1.2	4.00	a
80	KSC	Siam Queen OP	*O. basilicum*	k1	k1.2	4.00	a
81	Rutgers	RUMC_09	*O. basilicum*	k1	Admixed	4.00	a
82	Macedonia	PI 358465	*O. basilicum*	k1	k1.2	3.88	ab
83	Rutgers	Kivumbisi Lime x RUSB_17 F1	*O. americanum x O. basilicum*	k1	k1.2	0.00	s
84	Turkey	PI 170579	*O. basilicum*	k1	k1.2	3.63	abcd
85	Rutgers	Pesto Perpetuo	*O. basilicum*	k1	k1.2	3.13	abcdef
86	Rutgers	GCB	*O. basilicum*	k1	k1.2	1.38	klmnop
87	Rutgers	Pezou	*O. basilicum*	k1	k1.2	3.88	ab
88	JSS	Cinnamon1	*O. basilicum*	k1	k1.2	4.00	a
93	Turkey	PI 182246	*O. basilicum*	k1	k1.2	4.00	a

94	FS	Violetto Aromatico	*O. basilicum*	k1	k1.2	4.00	a
95	JSS	Purple Ruffles	*O. basilicum*	k1	k1.2	1.75	ijklmn
96	JSS	Red Rubin	*O. basilicum*	k1	k1.2	2.13	ghijkl
		PI 172996 x	*O. basilicum x*				
114	Rutgers	Sweet Dani F1	*O. citriodorum*	k2	k2.1	1.75	ijklmn
115	Rutgers	Sweet Dani	*O. citriodorum*	k2	k2.1	2.25	fghijk
116	Turkey	PI 172996	*O. basilicum*	k2	k2.1	0.63	pqrs
117	Turkey	PI 172998	*O. basilicum*	k2	k2.1	0.38	qrs
118	Iran	PI 253157	*O. basilicum*	k2	k2.1	1.88	hijklm
119	Iran	PI 296391	*O. basilicum*	k2	k2.1	0.63	pqrs
120	New Mexico	PI 652054	*O. basilicum*	k2	k2.1	2.13	ghijkl
121	EZ	Lemona	*O. citriodorum*	k2	k2.1	2.13	ghijkl
122	India	PI 652061	*O. basilicum*	k2	k2.2	2.88	cdefg
123	Pakistan	PI 652060	*O. americanum*	k2	k2.2	4.00	a
124	TSC	Lemon	*O. citriodorum*	k2	k2.2	3.13	abcdef
125	BCH .	Thai Hairy Lemon	*O. citriodorum*	k2	k2.2	4.00	a
126	KSC	Penang Lemon	*O. citriodorum*	k2	k2.2	3.00	bcdefg
127	BCH	Lime1	*O. americanum*	k2	k2.2	3.63	abcd
128	JSS	Lime2	*O. americanum*	k2	k2.2	3.13	abcdef
129	SOC	Lemon Heirloom	*O. citriodorum*	k2	Admixed	0.88	nopqrs
138	Rutgers	Blue Spice F1	*O. americanum*	k3	k3.1	0.00	s
139	RSS	Spice	*O. americanum*	k3	k3.1	0.00	s
140	Maryland	PI 414205	*O. tenuiflorum*	k3	k3.1	0.00	s

141	Maryland	PI 414204	*O. tenuiflorum*	k3	k3.1	0.00	s
142	Denmark	PI 652056	*O. tenuiflorum*	k3	k3.1	0.00	s
143	Maldives	PI 652059	*O. tenuiflorum*	k3	k3.1	0.00	s
144	Rutgers	Blue Spice	*O. americanum*	k3	k3.1	0.00	s
145	Maryland	PI 652052	*O. x africanum*	k3	k3.2	1.75	ijklmn
149	WSHF	Camphor	*O. kilidmandsharicum*	k3	k3.2	0.13	rs
150	Maryland	PI 652053	*O. basilicum*	k3	k3.2	0.25	rs
156	RSS	Holy Basil	*O. tenuiflorum*	k3	k3.2	1.00	mnopqr
157	India	PI 288779	*O. tenuiflorum*	k3	k3.2	0.00	s
158	RSS	Indian	*O. tenuiflorum*	k3	k3.2	0.13	rs
160	Brazil	PI 652066	*O. campechianum*	k3	k3.2	0.00	s
161	Zambia	PI 500945	*O. americanum*	k3	k3.3	0.00	s
162	Zambia	PI 500946	*Ocimum spp.*	k3	k3.3	1.00	mnopqr
163	Zambia	PI 500949	*O. x africanum*	k3	k3.3	0.00	s
164	Zambia	PI 500943	*O. x africanum*	k3	k3.3	1.88	s
165	Zambia	PI 500944	*O. x africanum*	k3	k3.3	0.00	s
166	Zambia	PI 500947	*O. x africanum*	k3	k3.3	1.13	s
167	Zambia	PI 500953	*O. x africanum*	k3	k3.3	0.00	s
168	Zambia	PI 500950	*O. x africanum*	k3	k3.3	0.25	rs
169	Zambia	PI 500954	*O. x africanum*	k3	k3.3	0.00	s
170	Zambia	ZA1	*Ocimum spp.*	k3	k3.3	4.00	a
171	Zambia	PI 500942	*O. x africanum*	k3	k3.3	0.25	s
172	Brazil	PI 652067	*O. gratissimum*	k3	k3.2	0.00	s

173	Brazil	PI 652068	*O. gratissimum*	k3	k3.2	0.00	s
174	Brazil	PI 652069	*O. gratissimum*	k3	k3.2	0.00	s
175	Zambia	PI 500952	*O. gratissimum var. gratissimum*	k3	k3.2	0.00	s
176	Taiwan	PI 211715	*O. gratissimum var. gratissimum*	k3	k3.2	0.00	s
177	SMS	Mtule	*Ocimum spp.*	k3	k3.2	0.00	s
178	Tanzania	PI 652064	*O. gratissimum var. gratissimum*	k3	k3.2	0.25	rs
179	Sri Lanka	PI 652055	*O. gratissimum var. macrophyllum*	k3	k3.2	0.00	s
180	SMS	Mzambda	*Ocimum spp.*	k3	k3.2	0.00	s
181	WSHF	Green Pepper Basil	*O. selloi*	k3	k3.2	0.63	pqrs
182	Uruguay	PI 511865	*O. selloi*	k3	k3.2		
89	Rutgers	RU172S_303	*O. basilicum*	Admixed	Admixed	3.50	abcde
90	Rutgers	RU172S_322	*O. basilicum*	Admixed	Admixed	3.75	abc
91	Rutgers	RU172S_306	*O. basilicum*	Admixed	Admixed	3.63	abcd
92	Rutgers	RU172S_315	*O. basilicum*	Admixed	Admixed	3.50	abcde
97	Rutgers	RUSB_22 x Lime F1	*O. basilicum x O. americanum*	Admixed	Admixed	3.88	ab
98	Rutgers	PI 652060 x RUSB_17 F1	*O. americanum x O. basilicum*	Admixed	Admixed	4.00	a

99	Rutgers	RU172S_01	*O. basilicum*	Admixed	Admixed	3.13	abcdef
100	Rutgers	SB17 x PI 172996 F1	*O. basilicum x O. basilicum*	Admixed	Admixed	2.75	defgh
101	Rutgers	PI 172996 x MRI F1	*O. basilicum x O. basilicum*	Admixed	Admixed	0.88	nopqrs
102	Rutgers	RU172S_05	*O. basilicum*	Admixed	Admixed	1.63	jklmno
103	Rutgers	Sweet Dani x RUMC_09 F1	*O. citriodorum x O. basilicum*	Admixed	Admixed	3.38	abcde
104	Rutgers	Sweet Dani x MRI F1	*O. citriodorum x O. basilicum*	Admixed	Admixed	0.75	opqrs
105	Rutgers	Sweet Dani x RUCB_10 F1	*O. citriodorum x O. basilicum*	Admixed	Admixed	3.38	abcde
106	Rutgers	RU172S_03	*O. basilicum*	Admixed	Admixed	2.63	efghi
107	Rutgers	RU172S_06	*O. basilicum*	Admixed	Admixed	1.25	lmnopq
108	Rutgers	RU172S_02	*O. basilicum*	Admixed	Admixed	2.25	fghijk
109	Rutgers	RU172S_04	*O. basilicum*	Admixed	Admixed	3.75	abc
110	Rutgers	RUSDCB_172 F2	*O. citriodorum x O. basilicum*	Admixed	Admixed	4.00	a
111	Rutgers	RUSDCB_174 F2	*O. citriodorum x O. basilicum*	Admixed	Admixed	3.50	abcde
112	Rutgers	RUSDCB_15101 F3	*O. citriodorum x O. basilicum*	Admixed	Admixed	3.75	abc

113	Rutgers	RUSDCB_151 F2	*O. citriodorum x O. basilicum*	Admixed	Admixed	3.63	abcd
130	Rutgers	Spice x PI 172996 F1	*O. americanum x O. basilicum*	Admixed	Admixed	0.00	s
131	Rutgers	Spice x Sweet Dani F1	*O. americanum x O. citriodorum*	Admixed	Admixed	0.00	s
132	Rutgers	Camphor x PI 172996 F1	*O. kilidmandsharicum x O. basilicum*	Admixed	Admixed	0.25	rs
133	Rutgers	MRI x Spice F1	*O. basilicum x O. americanum*	Admixed	Admixed	0.00	s
134	Rutgers	Spice x RUSB_09 F1	*O. americanum x O. basilicum*	Admixed	Admixed	0.00	s
135	Maryland	PI 414201	*O. tenuiflorum*	Admixed	Admixed	0.00	s
136	Maryland	PI 414203	*O. tenuiflorum*	Admixed	Admixed	0.00	s
137	Rutgers	Spice x DiGenova F1	*O. americanum x O. basilicum*	Admixed	Admixed	0.63	pqrs
146	Rutgers	African Blue Basil	*O. basilicum x O. kilidmandsharicum*	Admixed	Admixed	0.00	s
147	Rutgers	PI 172996 x Camphor F1	*O. basilicum x O. kilidmandsharicum*	Admixed	Admixed	0.31	rs
148	Rutgers	RUSB_17 x Camphor F1	*O. basilicum x O. kilidmandsharicum*	Admixed	Admixed	0.25	rs
151	Iran	PI 253158	*O. americanum*	Admixed	Admixed	0.00	s

152	Iraq	PI 254352	*O. americanum*	Admixed	Admixed	0.13	rs
153	SMS	Kivumbisi Lime	*O. americanum*	Admixed	Admixed	0.00	s
154	Togo	PI 652058	*O. americanum*	Admixed	Admixed	0.13	rs
155	SMS	African	*Ocimum spp.*	Admixed	Admixed	0.00	s
159	Cuba	PI 652057	*O. tenuiflorum*	Admixed	Admixed	2.25	fghijk
1	Rutgers	CR3	*Nepeta cataria*		Outgroup		
2	Rutgers	CR9	*Nepeta cataria*		Outgroup		

[a]Origin refers to the collection location reported by the United States Department of Agriculture National Genetic Resources Program (USDA-GRIN). Source refers to the following commercial seed companies:
[b]Species reported according to the source from which they were obtained
[c]Cluster and sub-cluster refers to the inferred population derived from primary and secondary (nested) model-based cluster analyses, respectively. Admixed accessions refer to those for which qI < 0.7 and /or accessions are from a a known bi-parental orgin of two different primary clusters.
[d]DS refers to disease severity determined by the extent of sporulation on the abaxial leaf surface
