# Supplementary material for: Population structure, genetic diversity and downy mildew resistance among Ocimum species germplasm
Source: BMC Plant Biol. 2018 Apr 23;18:69. doi: 10.1186/s12870-018-1284-7 (PMC5914031; doi:10.1186/s12870-018-1284-7)
Supplement: Supplementary file 2 — Primer sequences and melting temperatures (Tm) for the EST-SSRs used in this study. (PDF 52 kb) [file 12870_2018_1284_MOESM2_ESM.pdf]

Additional file 2. Primer sequences and melting temperatures (T_m) for the EST-SSRs used in this study.

Marker ID	NCBI / Contig ID	Forward Primer Sequence	T_m	Forward Reverse Sequence	T_m
OBNJR2sg33	DY333933	GCCTCTCCCTCCTCCATAAC	60.037	AGGCGACGAGCATGAAGTAG	60.559
OBNJR2cn29	Contig1138	TGGACATCAAATTGGCTTCA	60.049	TGGAAGGACTCGTCATCTCTC	59.395
OBNJR2sg04	DY343638	ACGATATGAGACATGGGCCT	59.39	CGCAGGTACAAGCTTCTCAA	59.22
OBNJR2sg30	DY336727	GCCAAATAATTCCTATCCGGT	59.202	CTTGGCTTTGGGAGATTCAC	59.67
OBNJR3sg124	DY331703	TAAGACAACAATCGGTGCCA	60.111	GTCCCATTCCTCCTCCGTAT	60.154
OBNJR3sg19	DY343509	AAGCCGCCCTATAAACCAAT	59.829	GGCCGTTACAAAGAGCTGAG	60.015
OBNJR3cn298	Contig2510	GACGCACCTCAAGAGTGATG	59.42	ATGGAACCATGGGAAGATGA	60.135
OBNJR3cn359	Contig2911	TAGGTCAGCTAGTGCGCAGG	61.654	GCAGAAGCGTATACATGCGA	60.006
OBNJR3cn362	Contig2969	GAAGAGATGGCTGGTCTTGG	59.803	AGACAGAGAGAGGGCAGCAG	59.883
OBNJR3sg155	DY325572	AATTCTCAGCAGGGTTGGTG	60.111	CAGCCTATTCGACGACAACA	59.864
OBNJR3sg168	DY323726	TTTATGAGATTGGCGCACAC	59.694	GCCATGTCCAGATCCTTGTT	59.934
OBNJR3sg113	DY335879	CAGATGACCACACCGAAATG	59.96	TGCAGTGAGAATGAAGGTGC	59.992
OBNJR3cn56	Contig582	GAAACAACATCCCTCATGCC	60.326	TTGAGATTGGGTTGGAGGAG	60.042
OBNJR3cn74	Contig715	GAAGGCGCTGAGAAGAAGAA	59.836	CCAATTCAACACAACCATCG	59.816
OBNJR3sg145	DY328393	GGAAATGTGGTCGTTATTCACA	59.73	CCAAAGGAAGCGACAATGAT	60.074
OBNJR3cn03	Contig100	CCGCTCTGATCTTCACTTCC	59.95	TTCACAGTCGATTCAGCAGG	59.984
OBNJR3cn210	Contig1890	CGATCATGGTGTCTCAGTGTG	60.17	GAGAGGTATCCGGTGCACAT	59.957
OBNJR3cn240	Contig2142	AACACACAAAGATCCAAACCC	58.803	TTATTTCCCAACCCACTACCA	59.188
OBNJR3sg13	DY344184	CAACAACAACGGAGAGCAAA	59.881	AAATACTTGCCGTTTGCGAC	60.138
OBNJR4cn17	Contig2461	TGAAGGCTTTGAAGAGGTAAAGA	59.548	TTCTGCTGGGCTTTGAGTTT	59.993
