# Supplementary material for: Population structure, genetic diversity and downy mildew resistance among Ocimum species germplasm
Source: BMC Plant Biol. 2018 Apr 23;18:69. doi: 10.1186/s12870-018-1284-7 (PMC5914031; doi:10.1186/s12870-018-1284-7)
Supplement: Supplementary file 3 — Structure Harvester output for primary model-based clustering using Structure ver 2.3.4 software. Plot of ΔK (left) and LnP(D) (right) for K = 1 – 15. (PDF 126 kb) [file 12870_2018_1284_MOESM3_ESM.pdf]

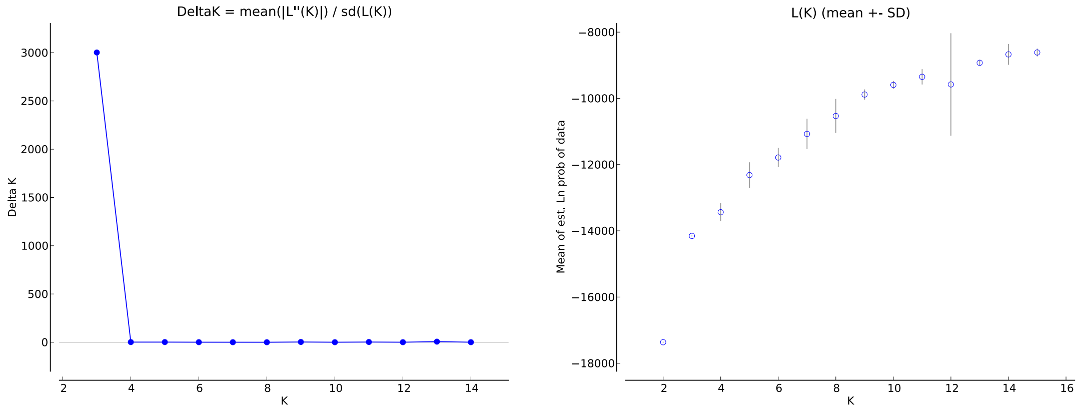

Additional file 3. Structure Harvester output for primary model-based clustering using Structure ver 2.3.4 software. Plot of Δ*K* (left) and LnP(D) (right) for *K* = 1 – 15.
