# Supplementary material for: Population structure, genetic diversity and downy mildew resistance among Ocimum species germplasm
Source: BMC Plant Biol. 2018 Apr 23;18:69. doi: 10.1186/s12870-018-1284-7 (PMC5914031; doi:10.1186/s12870-018-1284-7)
Supplement: Supplementary file 4 — Structure Harvester output for secondary (nested) model-based clustering using Structure ver 2.3.4 software. Plots of ΔK (left) and LnP(D) (right) for K = 1 – 10. Three nested analyses shown for k1 (top), k2 (middle) and k3 (bottom) primary clusters. (PDF 215 kb) [file 12870_2018_1284_MOESM4_ESM.pdf]

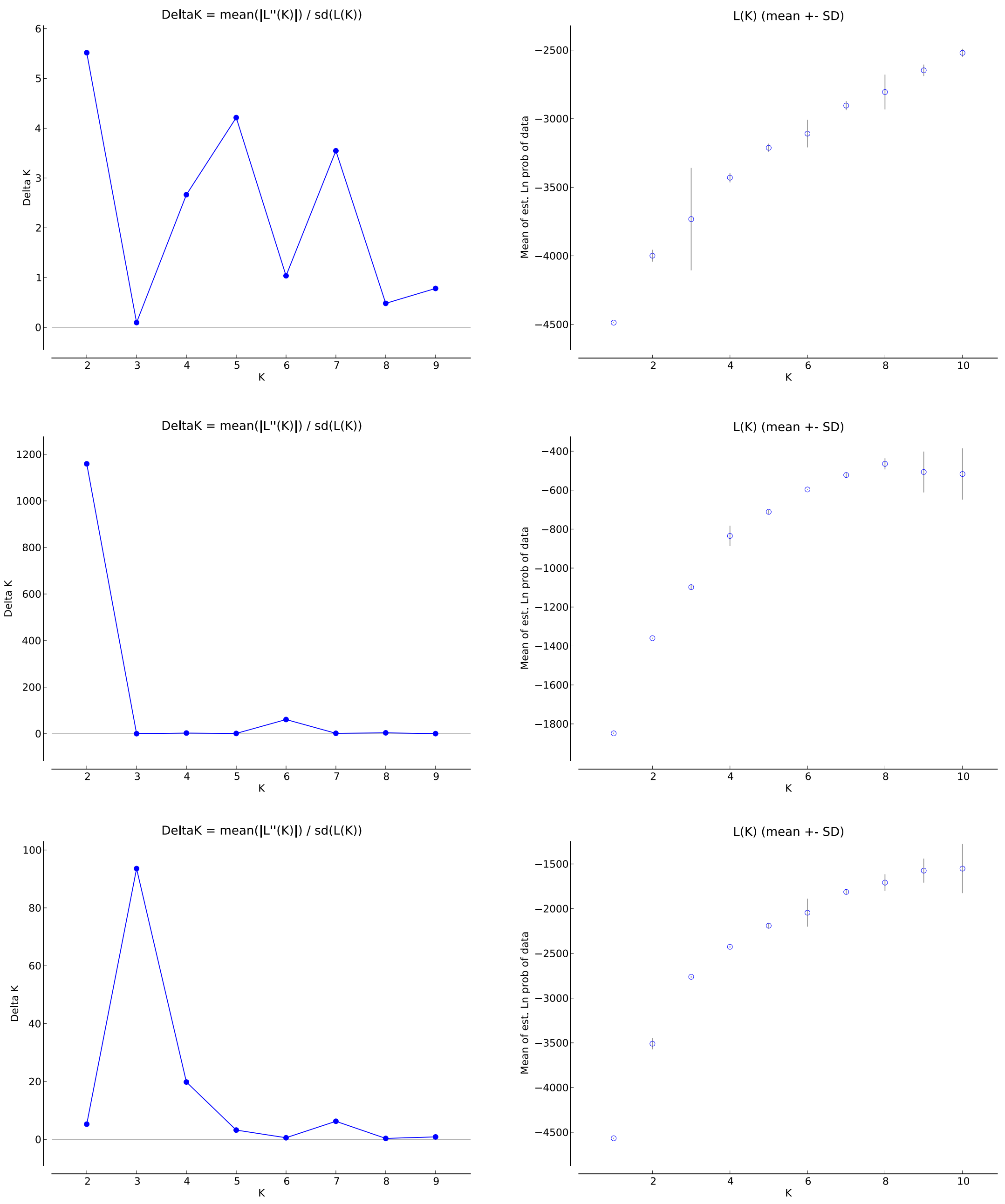

Additional file 4. Structure Harvester output for secondary (nested) model-based clustering using Structure ver 2.3.4 software. Plots of ΔK (left) and LnP(D) (right) for $K = 1 – 10$. Three separate nested analyses shown for k1 (top), k2 (middle) and k3 (bottom) primary clusters.
